# Mental health conditions and academic burnout among medical and non-medical undergraduates during the mitigation of COVID-19 pandemic in China

**DOI:** 10.1007/s11356-022-19932-2

**Published:** 2022-03-31

**Authors:** Qian Yang, Yueheng Liu, Winson Fuzun Yang, Pu Peng, Shubao Chen, Yunfei Wang, Xin Wang, Manyun Li, Yingying Wang, Yuzhu Hao, Li He, Qianjin Wang, Junhong Zhang, Yuejiao Ma, Haoyu He, Yanan Zhou, Jiang Long, Chang Qi, Yi-Yuan Tang, Yanhui Liao, Jinsong Tang, Qiuxia Wu, Tieqiao Liu

**Affiliations:** 1 Department of Psychiatry, and National Clinical Research Center for Mental Disorders, The Second Xiangya Hospital of Central South University, Changsha, 410011 Hunan China; 2 National Clinical Research Center of Mental Disorders, Changsha, Hunan People’s Republic of China; 3 Department of Psychological Sciences, Texas Tech University, Lubbock, TX USA; 4 Department of Psychiatry, Hunan Brain Hospital (Hunan Second People’s Hospital), Changsha, China; 5 Shanghai Mental Health Center, Shanghai Jiao Tong University School of Medicine, Shanghai, China; 6 Department of Psychiatry, Zhejiang Provincial People’s Hospital, People’s Hospital of Hangzhou Medical College, Hangzhou, Zhejiang People’s Republic of China; 7 College of Health Solutions, Arizona State University, Phoenix, AZ USA; 8 Department of Psychiatry, School of Medicine, Sir Run Run Shaw Hospital, Zhejiang University, Hangzhou, Zhejiang People’s Republic of China

**Keywords:** Academic burnout, COVID-19 pandemic, Mental health conditions, Undergraduate students

## Abstract

The outbreak of the novel coronavirus disease 2019 (COVID-19) has posed a great impact on people’s mental health, especially for undergraduate students. This study aimed to compare the mental health conditions and academic burnout between medical and non-medical undergraduates in China when the COVID-19 pandemic is mitigating. A cross-sectional online survey was conducted among 4,972 undergraduates between October 2020 and April 2021, when the pandemic was basically under control. The survey included basic demographics information and standardized scales to evaluate depression, anxiety, perceived stress, daytime sleepiness, alcohol abuse/dependence, quality of life, fatigue, and academic burnout. Compared with medical undergraduates, non-medical undergraduates had higher rates of moderate to severe depression symptoms (29.1% vs. 17.9%, *P* < 0.001), moderate to severe anxiety symptoms (19.7% vs. 8.9%, *P* < 0.001), alcohol abuse/dependence (16.3% vs.10.3%, *P* < 0.001), excessive daytime sleepiness (47.4% vs. 43.4%, *P* = 0.018), high perceived stress (34.7% vs. 22.2%, *P* < 0.001), high level of fatigue (51.8% vs. 42.2%, *P* < 0.001), low QOL (35.8% vs. 21.4%, *P* < 0.001), and higher academic burnout score (59.4 vs. 57.5, *P* < 0.001). Being non-medical undergraduates, depression, alcohol abuse/dependence, excessive daytime sleepiness, and high perceived stress were positively associated with academic burnout, while high QOL was negatively associated with the burnout (all *P* < 0.001). Excessive daytime sleepiness was the strongest predictor for academic burnout.

## Introduction

Mental health conditions have always been a hotspot of studies given their importance, especially for undergraduates. With the outbreak of the novel coronavirus disease 2019 (COVID-19) pandemic, governments around the world have taken effective measures to avoid its spread (Anderson et al. 2020). Undergraduate students are vulnerable in the face of the pandemic (Guessoum et al. 2020). To date, it has been reported that the COVID-19 pandemic has brought numerous adverse impact on the mental health of undergraduates, such as internet addiction, acute stress disorder, anxiety, depression, and insomnia (Marelli et al. 2021; Saddik et al. 2020; Shehata & Abdeldaim 2021; Ye et al. 2020; Yu et al. 2021). Furthermore, non-medical undergraduates reported much more mental health problems than medical undergraduates during the early stage of the COVID-19 pandemic (Xie et al. 2020). Poor mental health conditions will bring a huge burden to the society and economy (G. Sani et al. 2020a, b). As the coronavirus is susceptible to mutation, there is always risk for another outbreak or even long-term existence of the virus. Therefore, it is important to evaluate the mental health conditions among undergraduates, especially medical undergraduates, while the COVID-19 pandemic is subsiding.

Burnout is a syndrome characterized by emotional exhaustion, depersonalization, and reduced personal accomplishment (Rotenstein et al. 2018). In recent years, researchers have set their sights on burnout, and their attention slowly shifted from professionals to undergraduates, especially for medical undergraduates. A previous study showed that more than 50% of medical students in the USA are at risk of burnout (L. Dyrbye & Shanafelt 2016). With the popularization of higher education, the academic burnout among Chinese students is becoming more and more serious (Sufei et al. 2020). Numerous studies have found that academic burnout can lead to negative consequences, such as thoughts of dropout, alcohol abuse/dependence, and even suicidal ideation (L. N. Dyrbye et al. 2008; L. N. Dyrbye, et al. 2010b, a; Jackson et al. 2016). Meanwhile, academic burnout may cause negative consequences to occupational values of future work (Dall'Ora et al. 2020; L. N. Dyrbye, et al. 2010b, a; L. Dyrbye & Shanafelt 2016). Changes in learning patterns during the COVID-19 pandemic have increased the risk of academic burnout and affected academic performance as remote learning could reduce the communication between teachers and students (Sani et al. 2020b); however, whether the consequences would recover after the pandemic still remains unknown. Therefore, the academic burnout among undergraduates during the subsiding period of the COVID-19 pandemic is worthy of studying.

From 2002 to 2018, the number of students receiving higher medical training had increased by 8.2% to 182,900 (W. Wang 2021), which was substantial; however, only 15.91% of medical graduates registered as doctors over the past decade (Lien et al. 2016), and there is a serious lack of medical workers in China (Wu et al. 2016). Academic burnout and poor mental health conditions are highly likely to accelerate the loss of medical workers (Chen et al. 2021). A previous study reported that medical students suffered from a higher level of burnout than their non-medical counterparts (L. N. Dyrbye et al. 2014). In Chinese medical education system, the 5-year undergraduate medical education is only the entry level of clinical practice (W. Wang 2021). Of the 182,900 students who received higher medical education, which was mentioned above, 94,600 (51.7%) achieved a bachelor’s degree (W. Wang 2021). Thus, it is necessary to study the academic burnout and mental health conditions of this population, and compare their conditions with those of non-medical undergraduates if appropriate. The present study aimed to compare the mental health conditions and academic burnout between medical and non-medical undergraduates during the subsiding period of the COVID-19 pandemic and explored the relationship between mental health conditions and academic burnout. We hypothesized that medical undergraduates had better mental health conditions but a higher level of academic burnout compared to non-medical undergraduates. We also hypothesized that there might be a correlation between mental health conditions and academic burnout among undergraduates.

## Material and methods

### Participants and data collection

An online survey was conducted from October 2020 to April 2021, when the COVID-19 pandemic was basically under control in most areas of China. The online questionnaire was designed using Questionnaire Star (the most widely used online survey platform in China) and then sent to participants mainly via WeChat. The sample size was determined based on the mean standard deviation of previous studies. A total of 4,972 undergraduates volunteered to participate in the survey anonymously and provided written informed consent. All participants received immediate feedback on their mental health conditions and were provided with consultations with psychiatrists if they needed. Data cleaning involved review of data consistency and deletion of missing values, after which a total of 4,661 (93.74%) valid questionnaires were included in further analyses. This study was approved by the Ethics Committee of the Second Xiangya Hospital of Central South University.

### Study measures

We used established instruments to measure academic burnout and mental health conditions, including depression, anxiety, daytime sleepiness, perceived stress, fatigue, quality of life (QOL), and alcohol abuse/dependence. The survey also included questions about basic demographic information (age, gender, relationship status, academic year, and monthly income).

#### Academic burnout

Academic burnout is measured using the Academic Burnout Scale (ABS), a 20-item questionnaire that is valid and reliable in assessing academic burnout among Chinese undergraduates (Rong et al. 2005). The ABS includes three domains of academic burnout: dejection (8 items), improper behavior (6 items), and reduced personal accomplishment (6 items) (M. Wang et al. 2019a, b). In this study, the Cronbach’s alpha coefficients for the whole scale and each subscale were 0.90, 0.86, 0.77, and 0.78, respectively. Each item was graded on a 5-point scale (from 1 = “strongly disagree” to 5 = “strongly agree”) and the total score ranged from 20 to 100, with higher scores, indicating higher severity of academic burnout.

#### Depression and anxiety

The 9-item Patient Health Questionnaire (PHQ-9) and 7-item Generalized Anxiety Disorder Scale (GAD-7) were used to assess the symptoms of depression and anxiety (Kroenke et al. 2001; Spitzer et al. 2006), respectively, with higher scores indicating higher levels of depression and anxiety. Based on the scores, participants were classified as having normal (0–4), mild (5–9), and moderate to severe (≥ 10) depression/anxiety symptoms (Manea et al. 2012; Plummer et al. 2016).

#### Daytime sleepiness and perceived stress

Daytime sleepiness and perceived stress were assessed with the Epworth Sleepiness Scale (ESS) and the 10-item Perceived Stress Scale (PSS-10), respectively. Both scales have been used in previous studies and demonstrated good validities (Johns 1991; Z. Wang et al. 2011). The cutoff point of ESS was 11, with scores over 11 interpreted as excessive daytime sleepiness; the cutoff point of PSS-10 was 20, with scores over 20 interpreted as moderate and high levels of perceived stress (Shen et al. 2019; Zhan et al. 2021).

#### Alcohol abuse dependence

Alcohol abuse/dependence was measured by using the 3-item Alcohol Use Dependence Identification Test (AUDIT-C); it has the same sensitivity as the full-length AUDIT, as demonstrated in previous studies (Gual et al. 2002).

#### QOL and fatigue

All participants were required to rate their overall, mental, and emotional QOL over the past week on a standardized linear analogue scale (from 0 = “as bad as it can be” to 10 = “as good as it can be”). The validity of the scale has been established in previous studies (Rummans et al. 2006; West et al. 2011). Similarly, participants were required to rate their level of fatigue on a standardized linear analogue scale in the same way (where lower scores indicated higher level of fatigue). The participants with a score of 5 or lower were considered to have higher level of fatigue and low QOL (West et al. 2011).

### Statistical analysis

Independent samples *t* test, Chi-square test, and Fisher’s exact test were used to compare mental health conditions and academic burnout between groups when appropriate. Univariate linear regression analyses were used to determine the factors that were selected for the multiple regression model of mental health conditions and academic burnout. Multiple linear regression analyses combined with the stepwise forward method were used to explore the relationship between mental health conditions and academic burnout among the undergraduates. In the process of modeling, academic burnout was used as the dependent variable, with age, gender, academic year, relationship status, monthly income, student type, and mental health conditions as independent variables. All statistical analyses were conducted using the SPSS software 26.0. Two-sided *P* < 0.05 was considered statistically significant.

## Results

### Demographic characteristics of the participants

Among the 4,661 valid questionnaires, 3,473 (74.51%) were from medical undergraduates and 1,188 (25.49%) were from non-medical undergraduates. In this study, there were no significant differences between medical undergraduates and non-medical undergraduates in gender or relationship status (both *P* > 0 0.05, Table 1). Non-medical undergraduates were younger and had higher monthly income than medical undergraduates (both *P* < 0.001, Table 1). Furthermore, non-medical undergraduates were mostly freshmen, while medical undergraduates were mostly juniors.

**Table 1:** Demographic characteristics of medical undergraduates and non-medical undergraduates

Variables	Medical undergraduates (*N* = 3,473)	Non-medical undergraduates (*N* = 1,188)	*P* value
**Age, year**			< 0.001**
Mean (S.D.)	19.7 (1.47)	19.4 (1.53)	
**Gender (%)**			**0.735**
Male	1,085 (31.2%)	377 (31.7%)	
Female	2,388 (68.8%)	811 (68.3%)	
**Relationship status (%)**			**0.320**
Single	2,725 (78.5%)	910 (76.6%)	
Partnered	742 (21.4%)	277 (23.3%)	
Married	6 (0.2%)	1(0.1%)	
**Academic year**			< 0.001**
1st year	982 (28.3%)	478 (40.2%)	
2nd year	951 (27.4%)	201 (16.9%)	
3rd year	1,100 (31.7%)	327 (27.5%)	
4th year	307 (8.8%)	182 (15.3%)	
5th year	133 (3.8%)	0 (0%)	
**Monthly income, RMB/month**			< 0.001**
≤ 615	1,666 (48.0%)	472 (39.7%)	
616–1,310	1,071 (30.8%)	345 (29.0%)	
1,311–2,086	617 (17.8%)	275 (23.1%)	
2,087–3,270	94 (2.7%)	61 (5.1%)	
3,271–6,366	15 (0.4%)	20 (1.7%)	
> 6,367	10 (0.3%)	15 (1.3%)	

^*^Statistically significant (*P* < 0.05)

^**^Highly statistically significant (*P* < 0.01)

### Comparison of mental health conditions and academic burnout between medical undergraduates and non-medical undergraduates

Among the undergraduates, the prevalence of depression and anxiety symptoms was 64.9% and 45.8%, respectively. Compared to medical undergraduates, non-medical undergraduates were more likely to have moderate to severe depression symptoms (29.1% vs. 17.9%, *P* < 0.001), moderate to severe anxiety symptoms (19.7% vs. 8.9%, *P* < 0.001), high perceived stress (34.7% vs. 22.2%, *P* < 0.001), alcohol abuse/dependence (16.3% vs. 10.3%, *P* < 0.001), high fatigue (51.8% vs. 42.2%, *P* < 0.001), and low QOL (35.8% vs. 21.4%, *P* < 0.001) (Table 2). Furthermore, non-medical undergraduates showed a higher level of academic burnout (59.4 vs. 57.5, *P* < 0.001).

**Table 2:** Mental health conditions and academic burnout of medical undergraduates and non-medical undergraduates

Variables	Medical undergraduates (*n* = 3,473)	Non-medical undergraduates (*n* = 1,188)	*P* value
**Depression**			< 0.001**
Normal	2,850 (82.1%)	842 (70.9%)	
Moderate to severe depression	623 (17.9%)	346 (29.1%)	
**Anxiety**			< 0.001**
Normal	3,165 (91.1%)	954 (80.3%)	
Moderate to severe anxiety	308 (8.9%)	234 (19.7%)	
**Alcohol abuse/dependence**			< 0.001**
No	3,114 (89.7%)	994 (83.7%)	
Yes	359 (10.3%)	194 (16.3%)	
**Daytime sleepiness**			0.018*
Normal	1,964 (56.6%)	625 (52.6%)	
EDS	1509 (43.4%)	563 (47.4%)	
**Perceived stress**			< 0.001**
Normal to medium	2,703 (77.8%)	776 (65.3%)	
HPS	770 (22.2%)	412 (34.7%)	
**Fatigue (%)**			< 0.001**
Low	2,007 (57.8%)	573 (48.2%)	
High	1,466 (42.2%)	615 (51.8%)	
**Quality of life (%)**			< 0.001**
Low	743 (21.4%)	425 (35.8%)	
High	2,730 (78.6%)	763 (64.2%)	
**Academic burnout**			< 0.001**
Mean (S.D.)	57.5 (7.46)	59.4 (7.88)	

*EDS*, excessive daytime sleepiness; *HPS*, high perceived stress

^*^Statistically significant (*P* < 0.05)

^**^Highly statistically significant (*P* < 0.01)

### Association between mental health conditions and academic burnout

According to univariate linear regression analyses, all the factors of mental health conditions were significantly correlated with academic burnout. Therefore, we further explored the relationship between mental health conditions and academic burnout through multiple linear regression. After controlling for age, gender, relationship status, academic year, and monthly income, the multiple linear regression analysis showed that being non-medical undergraduates (*B* = 1.406; ***β*** = 0.091; 95% CI: 0.979–1.834, *P* < 0.001), moderate to severe depression symptoms (*B* = 2.134; ***β*** = 0.129; 95% CI: 1.585–2.683, *P* < 0.001), high perceived stress (*B* = 1.339; ***β*** = 0.086; 95% CI: 0.824–1.854, *P* < 0.001), excessive daytime sleepiness (*B* = 2.117; ***β*** = 0.156; 95% CI: 1.736–2.498, *P* < 0.001), alcohol abuse/dependence (*B* = 1.253; ***β*** = 0.065; 95% CI: 0.781–1.725, *P* < 0.001), and high level of fatigue (*B* = 1.516; ***β*** = 0.072; 95% CI: 0.930–2.101, *P* < 0.001) were positively associated with academic burnout, while high QOL (*B* =  − 0.703; ***β*** =  − 0.045; 95% CI: − 1.209 ~  − 0.196, *P* = 0.007) was negatively associated with academic burnout. Anxiety and fatigue were not selected by the stepwise forward regression model (Table 3).

**Table 3:** Relationship between mental health conditions and academic burnout (with age, gender, relationship status, academic year, and monthly income controlled)

	*B*	SE	*β*	*t*	*P* value	95% CI
NMU (vs. MU)	1.406	0.218	0.091	6.449	< 0.001**	0.979–1.834
MSD (vs. normal)	2.134	0.280	0.129	7.620	< 0.001**	1.585 − 2.683
HPS (vs. normal)	1.339	0.263	0.086	5.099	< 0.001**	0.824 − 1.854
EDS (vs. normal)	2.117	0.194	0.156	10.895	< 0.001**	1.736 − 2.498
AB (yes vs. no)	1.516	0.299	0.072	5.072	< 0.001**	0.930 − 2.101
QOL (high vs. low)	− 0.703	0.258	− 0.045	− 2.720	0.007**	− 1.209 − 0.196

*NMU*, non-medical undergraduates; *MU*, medical undergraduates; *MSD*, moderate to severe depression symptoms; *HPS*, high perceived stress; *EDS*, excessive daytime sleepiness; *AB*, alcohol abuse/dependence; *QOL*, quality of life; *SE*, standard error; *95% CI*, 95% confidence interval

^*^Statistically significant (*P* < 0.05)

^**^Highly statistically significant (*P* < 0.01)

## Discussion

To our knowledge, this is the first study to compare mental health conditions and academic burnout between medical undergraduates and non-medical undergraduates during the mitigating period of the COVID-19 pandemic. The survey showed that non-medical undergraduates had poorer mental health conditions and a higher level of academic burnout than medical undergraduates. Poor mental health conditions, more specifically, moderate to severe depression, alcohol abuse/dependence, excessive daytime sleepiness, and high perceived stress were positively associated with academic burnout; however, high QOL was negatively associated with academic burnout. These results call for effective interventions to improve undergraduate students’ mental health conditions and reduce academic burnout.

Our findings showed that mental health problems among undergraduates remain prevalent during the mitigating period of the COVID-19 pandemic. Among all the participants, 20.79% had moderate to severe depression symptoms, higher than 9% during the peak time of COVID-19 pandemic with the use of the same measurements and cutoff points (Tang et al. 2020). Our results were consistent with previous studies conducted on the general population, but we found a higher prevalence of depression during the mitigating period of the COVID-19 pandemic than that during the peak time of COVID-19 pandemic, suggesting that the pandemic might have a long-term impact on undergraduates’ mental health (Li et al. 2021; Shi et al. 2021). However, 64.9% of our participants have moderate to severe depression symptoms, higher than 33.6% among the general population during a similar period in China (Shi et al. 2021), indicating that undergraduates are more vulnerable to mental health problems. According to some previous studies, prolonged home quarantine, reduced face-to-face activities, excessive exposure to screens and smart devices, insufficient social support, and academic stress can cause mental health problems to undergraduates (Ma et al. 2020; Maras et al. 2015; Mheidly et al. 2020). The high prevalence of mental health problems during the mitigating period of the pandemic might reflect the ongoing psychological harm brought about by COVID-19, as the students still had academic stress and long screen time after returning to campus. Due to the need for prevention and control of the pandemic, most universities have to postpone the start of the semester, resulting in a backlog of courses. Besides, the combination of online and offline teaching methods increased screen exposure for students (Mheidly et al. 2020). Thus, a better study mode and stress coping strategy is needed to reduce screen time and academic stress for undergraduates.

Our results also showed that non-medical undergraduates had a higher prevalence of depression and anxiety than medical undergraduates, which was consistent with studies conducted during the peak time of the COVID-19 pandemic (Xie et al. 2020; Xiong et al. 2021), suggesting that medical undergraduates might be less affected by the pandemic or recover faster than non-medical undergraduates. With more knowledge about the pandemic, medical undergraduates tended to be less affected psychologically, as compared with non-medical undergraduates (Saddik et al. 2020; Xie et al. 2020). Previous study also showed that more knowledge of the COVID-19 pandemic was associated with less mental distress (Chang et al. 2020). In fact, it was previously believed that medical students might have more serious mental health problems than non-medical students, as they are often faced with high expectations patients, family members, and even themselves (Puthran et al. 2016). However, studies have found that medical students can better cope with stress using positive coping styles (Sitarz et al. 2021). Thus, for non-medical students, more knowledge about COVID-19 and positive coping styles are needed.

Our study showed that the level of burnout was very close to that found in previous studies (Xinyue et al. 2020), but surprisingly, being a non-medical undergraduate was significantly related to academic burnout. The result was unexpected, as previous studies showed that academic burnout was more severe among medical students than non-medical students (L. N. Dyrbye et al. 2014; Shad et al. 2015). Intensive medical courses and longer training periods (at least 1 year longer than that for non-medical students) might lead to academic burnout among medical undergraduates (Q. Wang et al. 2019a, b). However, the level of resilience in medical undergraduates was significantly higher, which might be the reason for their lower level of academic burnout (Cheng et al. 2020; Lasheras et al. 2020). This unexpected finding might also be attributed to the poorer mental health conditions of non-medical undergraduates. According to our findings, moderate to severe depression symptoms, alcohol abuse/dependence, excessive daytime sleepiness, high perceived stress, and low QOL were positively associated with academic burnout. Furthermore, the multiple linear regression showed that excessive daytime sleepiness was the strongest predictor for academic burnout. As sleep is vital for the restoration of energy and the preparation for further studying(Goldstein & Walker 2014; Hershner & Chervin 2014), daytime sleepiness might be an important factor for poorer stress management, cognitive dysfunction, and lower self-evaluation, as well as academic inefficiency (Akers et al. 2018; Killgore et al. 2008; Pagnin et al. 2014). There was also some evidence supporting our findings that mental health conditions were associated with academic burnout. Depression and burnout have overlapping manifestations (Bianchi et al. 2015; Golonka et al. 2019), making it difficult to distinguish the two conditions. Perceived stress and alcohol abuse/dependence were positively related to academic burnout, which was consistent with the results of previous studies (Eaves & Payne 2019; Jackson et al. 2016). Higher perceived stress indicated higher levels of emotional exhaustion and intention of quitting (Eaves & Payne 2019). QOL is a subjective evaluation of one’s social status, living standard, and psychological state (Y. Wang et al. 2020). According to our findings, low QOL might increase the risk of academic burnout, as those with higher QOL were more satisfied with their social status and psychological state (Y. Wang et al. 2020), which reduced their negative emotions and increased their sense of personal achievement. All of our results further demonstrated that mental health conditions and academic burnout among undergraduates, especially non-medical students, warrant much more concerns from education authorities.

There were several limitations of this study. First, this is a cross-sectional study, which precluded us from reaching a causal relationship between mental health conditions, academic burnout, and the COVID-19 pandemic. Thus, longitudinal studies are needed in the future to determine the causal relationship between academic burnout and mental health conditions. Second, the number of medical students included in this study was nearly three times than that of non-medical students, which might have introduced some biases. Finally, the data in the present study were obtained using self-reported scales, which might bring memory bias. Despite the above limitations, this study has distinct advantages. We have found that non-medical undergraduates were in a poorer mental health status and had higher levels of academic burnout, as compared with medical undergraduates, during the mitigating period of the COVID-19 pandemic. In addition, even with controlling for other variables, mental health conditions were significantly correlated with academic burnout. Hopefully, our findings could provide reference for the development of post-epidemic measures to improve the mental health conditions and reduce academic burnout of undergraduates, especially for non-medical undergraduates.

## Conclusions

During the mitigating period of the COVID-19 pandemic, the mental health conditions of non-medical undergraduates were poorer than that of medical undergraduates, and a correlation was found between mental health conditions and academic burnout. Future studies are needed to identify the causal relationship between mental health conditions and academic burnout, in order to provide a basis for the development of more targeted measures to improve mental health conditions and reduce academic burnout for undergraduate students.

## Data Availability

The datasets used and/or analyzed in the present study are available from the corresponding author on reasonable request.
